# Growing a Professional Network to Over 3000 Members in Less Than 4 Years: Evaluation of InspireNet, British Columbia’s Virtual Nursing Health Services Research Network

**DOI:** 10.2196/jmir.3018

**Published:** 2014-02-21

**Authors:** Noreen Frisch, Pat Atherton, Elizabeth Borycki, Grace Mickelson, Jennifer Cordeiro, Helen Novak Lauscher, Agnes Black

**Affiliations:** ^1^School of NursingFaculty of Human and Social DevelopmentUniversity of VictoriaVictoria, BCCanada; ^2^School of Health Information ScienceFaculty of Human and Social DevelopmentUniversity of VictoriaVictoria, BCCanada; ^3^Provincial Health Services AuthorityVancouver, BCCanada; ^4^eHealth Strategy OfficeFaculty of MedicineUniversity of British ColumbiaVancouver, BCCanada; ^5^Providence Health CareVancouver Coastal HealthVancouver, BCCanada

**Keywords:** social networking, social media, nursing, health services, research, education

## Abstract

**Background:**

Use of Web 2.0 and social media technologies has become a new area of research among health professionals. Much of this work has focused on the use of technologies for health self-management and the ways technologies support communication between care providers and consumers. This paper addresses a new use of technology in providing a platform for health professionals to support professional development, increase knowledge utilization, and promote formal/informal professional communication. Specifically, we report on factors necessary to attract and sustain health professionals’ use of a network designed to increase nurses’ interest in and use of health services research and to support knowledge utilization activities in British Columbia, Canada.

**Objective:**

“InspireNet”, a virtual professional network for health professionals, is a living laboratory permitting documentation of when and how professionals take up Web 2.0 and social media. Ongoing evaluation documents our experiences in establishing, operating, and evaluating this network.

**Methods:**

Overall evaluation methods included (1) tracking website use, (2) conducting two member surveys, and (3) soliciting member feedback through focus groups and interviews with those who participated in electronic communities of practice (eCoPs) and other stakeholders. These data have been used to learn about the types of support that seem relevant to network growth.

**Results:**

Network growth exceeded all expectations. Members engaged with varying aspects of the network’s virtual technologies, such as teams of professionals sharing a common interest, research teams conducting their work, and instructional webinars open to network members. Members used wikis, blogs, and discussion groups to support professional work, as well as a members’ database with contact information and areas of interest. The database is accessed approximately 10 times per day. InspireNet public blog posts are accessed roughly 500 times each. At the time of writing, 21 research teams conduct their work virtually using the InspireNet platform; 10 topic-based Action Teams meet to address issues of mutual concern. Nursing and other health professionals, even those who rated themselves as computer literate, required significant mentoring and support in their efforts to adopt their practice to a virtual environment. There was a steep learning curve for professionals to learn to work in a virtual environment and to benefit from the available technologies.

**Conclusions:**

Virtual professional networks can be positioned to make a significant contribution to ongoing professional practice and to creating environments supportive of information sharing, mentoring, and learning across geographical boundaries. Nonetheless, creation of a Web 2.0 and social media platform is not sufficient, in and of itself, to attract or sustain a vibrant community of professionals interested in improving their practice. Essential support includes instruction in the use of Web-based activities and time management, a biweekly e-Newsletter, regular communication from leaders, and an annual face-to-face conference.

## Introduction

### InspireNet

In 2009, “InspireNet*”* (Innovative Nursing Services and Practice Informed by Research and Evaluation Network) was launched with the purpose of increasing nurses’ capacity for and interest in health services research in British Columbia (BC), Canada. Funded as part of the BC Nursing Research Initiative through the Michael Smith Foundation for Health Research, the network team accepted a mission to serve nurses and other health professionals in diverse and geographically dispersed settings. Operating a network virtually, with the ability to connect people both synchronously and asynchronously, was the only viable option to reach, engage, and support nurse clinicians, managers, educators, researchers, and students, given the nature of their lives and work. InspireNet was, to the planning team’s knowledge, the first health care professionals’ network in BC that attempted to conduct its work almost exclusively through Web 2.0, social media, and Web conferencing technologies, and likely one of the first of its kind in Canada. For the purpose of this paper, Web 2.0 is used in a broad sense to represent interactive forms of connectivity that permit many-to-many communications in both synchronous and asynchronous timeframes.

In 2012, preliminary findings documenting the network’s growth and the experience of one of our topic-based Action Teams were presented [1]. In this current paper, we present evaluative data gathered over a three-year period on network growth, the parts of the network that support frequent use of the network platform, the establishment of password-protected electronic communities of practice, or eCoPs, (some of which are “open” to all network members and others that are “closed” and available only to specific members of working teams), and a summary of data obtained from our surveys and interviews, indicating the reasons individuals joined and participate in the network.

### Review of the Literature

There is a rapidly developing body of literature on the use of Web 2.0 and social media in health. A Medline search indicates the number of publications have more than doubled over the past five years with more than 3500 related publications. Many of the papers discuss how electronic tools assist individuals to support their health and health conditions. Another group of papers focuses on the use of social media to enhance communication between health care providers and patients, and among health care providers. A gap was identified as a small but emerging area of research at the intersection of social media, Web 2.0, and health professional practice; there were 93 papers published, 67 of them within the last 5 years [1]. This research area focuses on how health care professionals use electronic tools to connect with and learn from one other and seeks to understand the impact of these professional virtual connections. Researchers have reported that social media can be an effective tool to establish mentoring relationships [2], serve as a teaching tool for continuing professional education [3,4], provide a means to share professional ideas [5,6], and serve as a way for professional organizations to reach out to members/potential members to broaden their scope [7]. In 2009, writers for the Medical Library Association News commented that “...never before have there existed so many opportunities to ‘meet’ other professionals...Social networking enables meetings and collaborations on a level that has never existed before…” [8]. A recent study conducted in the Netherlands indicates that nearly 60% of health care providers use social media professionally and that their motives for using it include increasing their knowledge, efficiencies, and communication with both patients and colleagues [9]. InspireNet’s use of Web 2.0 and social media adopted these ideas and extended the idea of professional networking through development of a formal virtual network, inviting nurses and other health care professionals to join and work collaboratively on issues or topics of personal interest. Further, the network espoused the view that eCoPs could provide a means for productive work to take place. InspireNet was developed as a network of electronic communities of practice connected to each other through a virtual platform with a common vision of supporting and using health services research.

Communities of practice, or CoPs, were initially described by authors influenced by social learning theory. Wenger identifies that learning takes place within social relationships and is not simply based on knowledge development [10]. In a CoP, people who share an interest in a topic, have a common concern, or wish to solve a problem, collaborate and share ideas and experiences, expanding their knowledge and expertise. A CoP takes time to develop, yet sustains itself as an entity working toward a common goal. There are “life-cycle” phases of CoPs describing a progression from being an informal group to an actively engaged community: (1) potential—an informal group, (2) coalescing—establishing a group identity, (3) maturing—actively working together to a shared goal, (4) stewardship—sustaining momentum, and (5) transformation—having accomplished a goal, the members then identify a new goal or disband [11]. eCoPs are communities making use of electronic, or virtual, platforms. Experiences of those using eCoPs demonstrate that factors such as voluntary involvement, distributed leadership, shared identity, transparency, accessibility, and being problem-focused contribute to their success [12]. Further, in a recent article published in this journal, the author commented that building and sustaining effective eCoPs requires an “enabler” and “strategic community management” [13].

One aspect of CoPs that has been described is the division of members into “core” and “peripheral” categories. These terms were first used by Lave and Wenger when they described those who were entering learning communities as “new learners”, standing in the background [14]. Over time, those peripheral members become more involved, becoming “core” members. As CoPs entered the Web-based world, authors noted that eCoPs seem to experience a similar dynamic. In eCoPs especially, the term “peripheral members”, or “lurkers”, emerged as referring simply to those who “look in” to the eCoP but are not active. At least one group of authors cautions against the use of this derogatory term, as “lurkers” may be not only passively benefitting from the eCoP activities, they may also become quite active or even core members when the work on the topic of interest speaks directly to them [15]. In Web-based eCoPs, peripheral members may be a majority of members, yet their motivations for membership, their commitment to the eCoP, and the benefits they accrue are difficult to measure.

### Background on InspireNet

#### Overview

InspireNet was designed to make full use of Web technologies with the goals to (1) support the professional development of nurses and other professionals in the area of health services research, (2) support the development of research teams, and (3) enhance knowledge translation within health care systems. There is no monetary cost for membership in the network and membership is completely voluntary. InspireNet accepted the notion from the beginning that there would be core and peripheral members and also that members might experience periods of greater or lesser participation, depending on multiple factors such as time constraints and the topic of discussion. InspireNet’s virtual platform is comprised of several components: a Web 2.0 website, Web conferencing, and social media. Each is described below.

#### The Website

**Figure 1:**
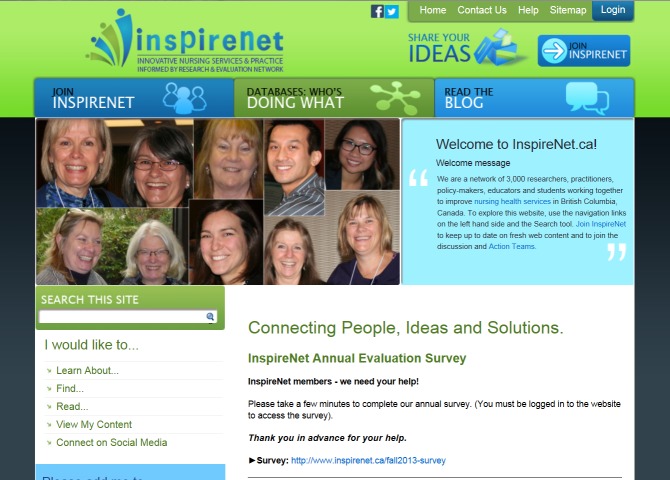
InspireNet home page.

**Figure 2:**
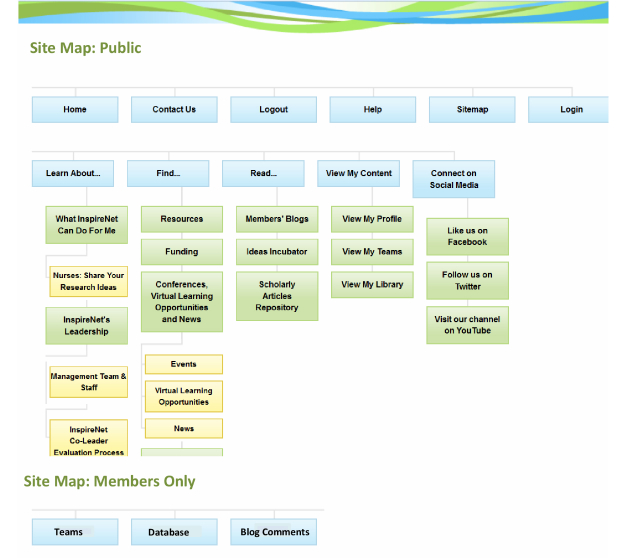
InspireNet site map.

The InspireNet website [16] was designed to be interactive and engaging, providing a platform for members to contribute to website content without advanced skills or specialized training. Figure 1 provides a screen capture of the site’s homepage. Figure 2 illustrates the website map. See Multimedia Appendix 1 for website details.

#### Web Conferencing

Supported by Cisco WebEx, Web conferencing provides InspireNet members the ability to connect in real time via their own computers, eliminating the need for travel and its associated burden. Action Teams use WebEx to facilitate for their team members free learning activities (webinars), discussions exploring potential research projects, and project development meetings. Closed Teams use WebEx in project development meetings, in conjunction with the website, for document-sharing and asynchronous discussion via forums within their eCoP. Web conferencing provides the ability for all teams to access guest speakers and consultants for lectures and discussions with team members. InspireNet teams have interacted with guests from across Canada and from Australia, Europe, and the United States. Action Teams’ webinars are recorded and archived for viewing by team members. At the time of writing, 79 webinars had been held with approximately 4000 views; InspireNet’s experience indicates that most views occur asynchronously via recordings, at members’ leisure.

#### Social Media

InspireNet leverages the power of social media to link relevant content with members, to grow membership, and to raise awareness about nursing health services research through an active Twitter feed (@InspireNetBC), a Facebook page (InspireNet), and a YouTube channel (InspireNetBC). Members are encouraged to include their LinkedIn account as a link in their database profile to advance connections using that platform.

### Distributed Leadership as a Working Model

**Figure 3:**
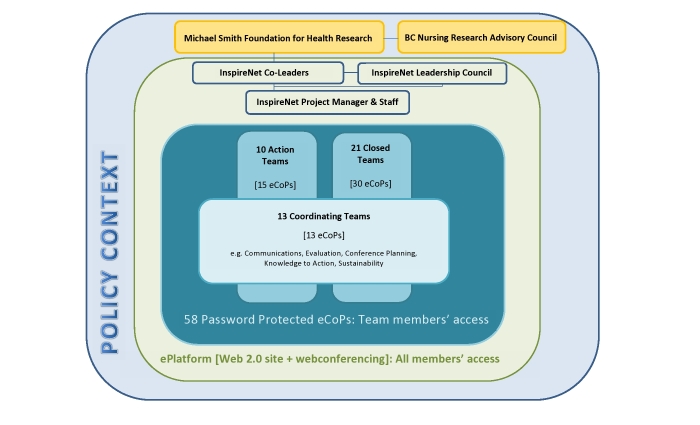
Network framework.

InspireNet does not rely on technology alone to support its members. A distributed leadership model was developed to promote and sustain member collaboration (see Figure 3). The distributed leadership model is innovative and has been a key part of the success of InspireNet. This model describes that having professionals work together creates collaborative advantages helpful to all [17]. The management team consists of three individuals—two co-leaders, one from clinical practice and one from academia, and a professional network manager. The network manager is retained at 0.8 full-time equivalent (FTE); the network co-leaders provide in-kind support to the network through their respective organizations that have agreed to serve as the network’s host institutions. With the exception of technical and scheduling support work carried out by the manager and a 0.2 FTE administrative support, all contributions to the network are voluntary. Coordinating teams’ members are professionals who volunteer to support the infrastructure and the work of the network. As is the nature of content on the World Wide Web generally and consistent with the theory on CoPs, InspireNet teams regulate themselves and contribute out of a desire to make a difference in their work and to support a common goal. Figure 3 provides an illustration of the relationship between the teams, where the Coordinating Teams support the work of Action and Closed Teams and the general membership.

## Methods

### Evaluation Framework

The network evaluation framework includes methods of retrieving data to document network activities, to understand the benefits and challenges of establishing a virtual professional network, and to learn what factors are valued and needed to support network growth. The range of evaluative methods for documenting and tracking members’ perceptions and usage of InspireNet are described in detail below.

### Network Growth

Monthly metrics are tabulated and reported as a dashboard and uploaded to the website for public access, demonstrating network growth generally, as well as in Action and Closed Teams, and milestone achievements.

### Network Use

Google Analytics is used by the network manager for the dashboard, reporting on webpage hits; periodically, more in-depth analysis is done to examine trends in website traffic in order to inform future website content decisions and to learn more about members’ use of the website. Additionally, Drupal provides metrics on the number of reads on each webpage for “on-the-fly” updates; these metrics are publicly available to any website visitor.

### Member Surveys

Network members have been surveyed twice. Online surveys addressed members’ satisfaction measured against achievements toward the network’s goals. Surveys were administered after 17 months of operation (Spring 2011) and after 30 months of operation (Spring 2012). Data were analyzed through descriptive statistics.

### Interviews

Interviews were conducted with Action Team leaders and members between 22 and 24 months of network operation (September to December 2011). Participants were selected via purposive sampling from those who indicated interest in participating in an interview. A protocol was developed to question interviewees about their involvement in InspireNet*/*Action Teams and their perceptions of the network’s activities and successes in reaching its goals. Interviews were 30-60 minutes, conducted by a graduate student via computer using Audacity (open source audio recording software), and transcribed verbatim. A coding scheme based on the framework of the InspireNet goals was developed by the evaluation team and NVivo (qualitative data analysis software) was used to code the 23 transcripts. Coding was done by four evaluation team members and a reliability check was conducted to ensure consistency across the transcripts. Once coded, the content was analyzed and summarized into themes based on ongoing discussions and feedback between evaluation team members.

### Stakeholder Feedback

At 33 months (July 2012), Action Team leaders were asked to submit brief narrative reports on their team’s activities, successes, challenges, and future plans. In total, 8 out of 10 Action Teams submitted reports, reflecting the team leaders’ perceptions and opinions; these data provided rich depictions for InspireNet leaders to better understand the internal factors for success within a team. At 36 months (October 2012), a workshop was held to engage key stakeholders in discussions about InspireNet’s successes and challenges as they related to network financial sustainability. The workshop included 15 members in person and 4 members participating via WebEx for a total of 19 participants working in small discussion groups. The consequent report is available at [18].

### Network Manager/Staff Reports

Annual reports to the funder and to the network’s Advisory Council provide a record of activities, member requests, challenges, and the management team’s responses to nurture and sustain the network. These reports provide a “behind-the-scenes” account of network activity and provide data on the work completed to support network functions.

## Results

### Report of Evaluative Data

#### Network Growth

**Figure 4:**
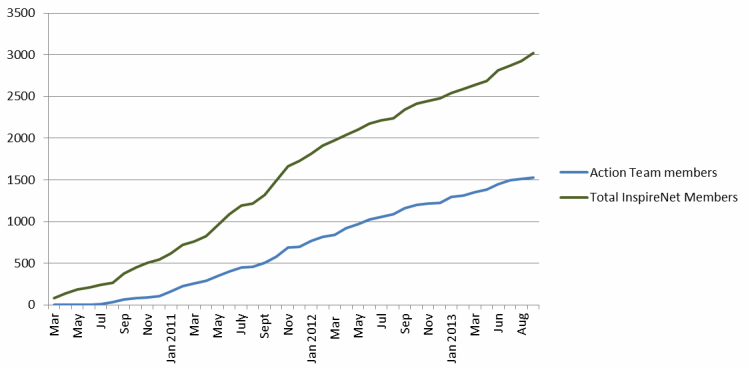
Action Team membership in relation to overall membership.

InspireNet’s growth continues to increase. At 47 months in the network’s life, there are over 3000 members involved to varying degrees with network activities (see Table 1). There are a total of 1442 members participating in Action Teams (see Figure 4). In total, 65.00% (1963/3020) of members work in clinical practice, 25.00% (755/3020) work or study in post-secondary educational institutions, and 10.00% (302/3020) work in smaller organizations and NGOs.

**Table 1:** InspireNet membership growth.

	12 months, Oct 2010	24 months, Oct 2011	36 months, Oct 2012	47 months, Sep 2013
No. of members	449	1484	2412	3020
% increase	-	231%	63%	25%

#### Website Use

Since its launch in May 2010 until September 2013, the website had received more than 76,000 hits with over 370,000 page views by more than 35,000 unique visitors, with an average of 5 pages at over 6 minutes per visit. Highest traffic times occurred immediately following the distribution of InspireNet’s biweekly eNews, a digest of all newly curated and hyperlinked website content. The most heavily accessed areas of the website were the teams’ eCoPs, followed by InspireNet’s “About Us” page, followed by the database. Interestingly, the database had been accessed on average 10 times per day. Of the publically accessible pages, the blogs posts had each been read on average more than 500 times.

Visitors resided primarily in Canada (87.00%, 30,450/35,000) and the United States (5.00%, 1750/35,000). Visitors from other countries resided in the United Kingdom, India, Australia, Philippines, and New Zealand.

Topic-based Action Team eCoPs increased their traffic steadily over time. Since their launch, the number of hits has increased markedly (see Table 2).

**Table 2:** Cumulative hits for Action Teams’ eCoPs.

Action Team^a^	12 months, Oct 2010	24 months, Oct 2011	36 months, Oct 2012	47 months, Sep 2013
eHealth, eTechnologies and Informatics	159	1060	2033	2848
First Nations Health	n/a	16	218	402
Healthy Workplace Climates	90	890	2992	4547
Initiative for a Palliative Approach in Nursing: Evidence & Leadership	n/a	n/a	1214	2017
Interdisciplinary Public Health Club	n/a	n/a	n/a	60
Nurse Educators’ Scholarship	206	1839	3687	5047
Nursing Education and Research Rounds	24	1526	3689	5026
Optimal Utilization of Advanced Practice Nursing Roles	262	1485	2041	2100
Practice-based Research Challenge	n/a	93	286	343
Students	n/a	n/a	79	337
Transition of New Grads	n/a	n/a	n/a	531
Total Action Teams’ eCoP Hits	741	6909	16,225	23,244

^a^n/a: not applicable where metrics are for a period that is prior to the launch of the team.

#### Survey Data

##### Survey #1

The respondent demographics mirrored those of the overall membership: 63.0% (443/703) of the overall membership and 56.0% (70/125) of the respondents worked in clinical practice settings; 29.9% (210/703) of the overall membership and 22.4% (28/125) of the respondents worked in post-secondary educational institutions; and the rest worked in government, NGOs, or other settings. No respondents identified themselves as novice computer users and a majority (69.6%, 87/125) rated themselves as average or proficient computer users. In total, 94.4% (118/125) reported that use of the Internet was useful or very useful to support their work activities, 64.5% (60/93) of the practitioners and managers noted that they had become more interested in embedding nursing health services research evidence in policy and practice as a result of their involvement in InspireNet, and 33.3% (14/42) of practitioners reported that they had changed their practice as a result of learning via the network. Furthermore, 35.7% (15/42) joined one or more of the topic-based Action Teams. Specifically related to network growth, 50.4% (63/125) of respondents reported becoming aware of the network through word of mouth communications and more than 50% (52.0%, 65/125) cited opportunities to network, learn, and collaborate as reasons for becoming a member. Also, 40.8% (51/125) indicated that they were learning new knowledge and skills, primarily related to research through their network membership and 43.2% (54/125) reported that they had or were currently working on research proposals with people they had met through InspireNet. The survey report is available at [19].

##### Survey #2

The demographic characteristics of the network and the survey respondents remained similar to those in the first survey: 61.0% (128/210) of the survey respondents worked in the health sector, 23.8% (50/210) in post-secondary educational institutions, and the rest in government, NGOs, or other areas. Geographic representation was likewise similar to the first survey response. Most respondents had been network members for one year or more. At this point, 85.2% (179/210) responded that they believed the network added value to provincial research capacity, 81.0% (170/210) responded that InspireNet provided good access to information, and 71.9% (151/210) responded that the network was promoting research in the province. In addition, 18.1% (38/210) of the respondents indicated that they had articles or research papers either published or in the process of development with individuals they had met through the network. Similar to responses from the first survey, a majority (73.8%, 155/210) of the respondents indicated that they had learned about the network through word-of-mouth communications. At this time, survey respondents began commenting on their need for discretionary time to work in a virtual environment and the skills they needed to learn to work virtually. Respondents’ written comments described a distinct learning curve with virtual working, particularly use of Web 2.0 interactive technologies. While the majority embraced this learning, some found it challenging. There was a general sense that participants considered virtual working to be the way of the future. The survey report is available at [20]. See Table 3 for a summary of survey activities.

**Table 3:** Survey data collection.

Survey	Time	Length	No. of reminders	No. of members	No. of respondents	Response rate
1	Spring 2011	4 weeks	3	703	125	17.78%
2	Spring 2012	6 weeks	6	2038	210	10.30%

#### Member Interviews

In total, 23 members, some of whom were Action Team leaders, were interviewed. The majority of those interviewed were active in InspireNet, being members of 2 or more Action Teams. Interview data provided findings about the network’s virtual platform, helping managers to better understand what was of perceived value. Interviews revealed that members benefited from the network in multiple ways, in terms of building research capacity, connecting and communicating with other professionals, and supporting their practice.

The theme of communicating with others to support one’s work and learning was prominent throughout the interviews, as illustrated by the following quotes:

I have learned a tremendous amount through InspireNet in the area [of] building capacity for health services research, knowledge translation, using an eCoP, and website to support the activities of a research team.

[The Action Team] brings everyone to my doorstep.

The resources, partnerships, and community of nurse-researchers brought together by InspireNet are incredibly helpful to me in my role supporting nursing research.

We need to talk to one another regularly and share resources and talk about how we overcame particular problems with [our] role and just checking in with each other. So [the eCoP has] been an absolutely vital communication link.

#### Stakeholder Feedback

Action Team leaders identified the value of webinars to provide educational opportunities, while noting the need for dedicating time to take on facilitation of their teams in their volunteer roles. Stakeholder workshop participants expressed their perceived value in InspireNet’s full suite of activities, recommending expanding the scope of the network to beyond the discipline of nursing and exploring ongoing funding options. Last, some stakeholders raised concerns about the usability of the network for users who were not accustomed to working virtually.

#### Manager/Staff Data

The network management team, in preparing annual reports, evaluated their focus of activities, their requests from the membership, and the problems encountered for each of the three years reported here. The following describes salient areas of growth and learning that emerged from this internal review.

In the first year of the network, the network manager spent considerable time in an educator role to teach members how to work virtually. These health care professionals, while experienced users of computers, did not have experience working in a virtual community, so that lessons, mentoring, and practice with webinars, becoming part of a virtual team, entering into synchronous and asynchronous discussions, and accessing wikis and blogs were necessary. The Action Team leaders needed additional education on leading Web-based discussions, recording them, and teaching others the typical protocols for Web-based conferencing and collaborating.

Further, during the first year, there were issues related to network access and use of public/private websites. One prominent issue was that of firewalls that some new members encountered with their work computers that prohibited access. Another was the need for all members to be educated on copyright protection relating to protected materials they were eager to share with others. Both of these issues were addressed, the first through conversations and actions at institutional levels and the second with a focused educational outreach on copyright legislation in our jurisdiction and directions to help members learn how to access copyrighted articles through the existing provincial health care library system.

During the second year, other issues arose. Some members provided feedback that the website was difficult to navigate. The management team arranged for a faculty member leading a group of students in a University of Victoria School of Health Information Science course to complete a usability evaluation of the website so that improvements could be made. Other issues in the second year were a result of network growth; the need for instruction on working in a virtual environment continued.

In its third year, the network developed Closed Team eCoPs for established research teams to conduct their work. These team leaders and members required orientation to the network tools and, in some cases, training to adapt them to their own work.

InspireNet has held an annual conference for each of its three years. The conferences have been attended by over 450 people and have provided a venue for continuing education, face-to-face networking, and for members to present their own research to each other. Conference evaluations reported that participants found value in this networking time and appreciated the opportunities to learn from one another. Overwhelmingly, participants reported a high level of satisfaction with these events.

### Cumulative Results

**Figure 5:**
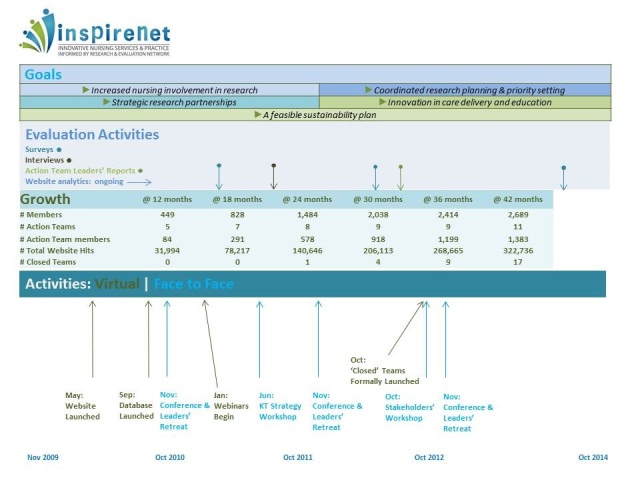
Timeline of growth and activities, June 2013.

Scrutiny of network growth patterns shows us that the network draws new members at the time of the annual conference, that hits on the website coincide with the publication of the biweekly eNewsletter, and that a majority of members entered into the network community after attending or retrieving a recording of a network Action Team webinar. Figure 5 indicates network growth in relation to other network activities, including website traffic, which may be an indicator of active membership.

## Discussion

### Principal Findings

#### Challenges in Evaluating a Virtual Network

For a network requiring active member participation and largely volunteer leadership, InspireNet has struggled to interpret the low rate of response to its annual evaluation surveys. While conclusions cannot be drawn about the ratio of core members versus peripheral members, network leaders question whether the ratio of core members to peripheral members described in other Web-based groups may be reflected in InspireNet, exhibited by a 10% to 18% response rate to surveys. It cannot be surmised that those who choose not to respond to surveys failed to respond because they are not actively engaged in the network (peripheral), are not getting something out of their network membership, or for some other reason. Further research in the area of active versus peripheral membership in Web 2.0 networks is needed.

#### Factors for Success in a Virtual Network

InspireNet has connected people across professional roles, academic settings, and geographical boundaries. These connections have fostered the formation of research partnerships and other collaborative work across the province; the technology used by InspireNet has enabled the creation of a network of eCoPs and has been critical to the achievement of the network’s goals. Experience of those using eCoPs documents that factors such as voluntary involvement, distributed leadership, shared identity, transparency, accessibility, and being problem-focused have contributed to their success [12]. Further, a recent case study of a professional network for dental care providers describes very similar challenges as those faced by InspireNet [21]. These authors identified the need for a regular schedule of activities in the eCoP, and the need for IT and user support. In addition, these authors acknowledge that while there are obvious benefits of having researcher-practitioner membership in professional eCoPs, there are challenges in terms of making activities relevant to both groups at once. The InspireNet experience is very consistent with these reports and, further, InspireNet has learned that active participation of a network management and staff team serving in a facilitator role is imperative for growth of the network and for welcoming and teaching new network members.

### Conclusions and Recommendations

InspireNet’s evaluation activities are ongoing, including a social network analysis exercise to better understand how the network operates. Network leaders are encouraged by the strength of membership numbers, which came as a surprise as the network has evolved, and by the outcomes that have been achieved in the first three years of the network. One factor that has proved essential to network growth is the presence of an active, responsive, and supportive network manager and the development of individuals in volunteer leadership roles who meet members’ needs to learn how to work in a virtual environment and also serve to welcome each new member. Evaluative data indicates that InspireNet grew because of word-of-mouth communications, that members join to connect with others, and that they increase their contact with the network immediately after network outreach activities. We conclude that InspireNet is meeting members’ needs for professional development, social interaction, and support, consistent with what is known about eCoPs and social learning. Because this is a network primarily of nurses, it would be of interest to explore whether this desire for professional social networking is a characteristic of the nursing population and whether or not this characteristic extends to other health care professionals. Further research on this topic is recommended.

Another lesson is the emerging role of “community manager”, which is evolving in the field of social media [13]. This role is unique, requiring leaders to facilitate and encourage online participation of community members. Recognizing that a majority of members entered Action Teams through webinars facilitated by team leaders, and remained active because of facilitated discussions on topics of interest, it is believed that having individuals step into this role as network Action Team leaders is a necessary part of both attracting and sustaining members. Last, InspireNet’s experience indicates that development of network tools alone is not sufficient for the growth and success of a professional network. A network team that communicates regularly with members as well as providing education, support, and evaluation of network activities is necessary to grow and support a network.

## Multimedia Appendix 1

InspireNet's website details.

## Abbreviations

eCoP: electronic communities of practice
FTE: full-time equivalent
InspireNet: Innovative Nursing Services and Practice Informed by Research and Evaluation Network
