# Supplementary material for: Growing a Professional Network to Over 3000 Members in Less Than 4 Years: Evaluation of InspireNet, British Columbia’s Virtual Nursing Health Services Research Network
Source: J Med Internet Res. 2014 Feb 21;16(2):e49. doi: 10.2196/jmir.3018 (PMC3961696; doi:10.2196/jmir.3018)
Supplement: Supplementary file 1 [file jmir_v16i2e49_app1.pdf]

The website was developed using Drupal, an open source content management system, which provides functionality that allows public access as well as password-protected areas available only to those individuals that have registered as network members.

Publically-accessible website content includes: general information about *InspireNet*; a blog which, as a knowledge translation vehicle, is written by various network members highlighting their areas of expertise and research projects; links to learning tools, resources related to nursing health services, and forthcoming conference listings with abstract deadlines for members' knowledge translation needs; pod/webcasts for skill-development; an archive of relevant nursing health services-related news; and links to *InspireNet*'s Twitter feed, Facebook page, and YouTube channel.

Password-protected areas of the website, available only to *InspireNet* members, provide access to both *InspireNet*'s member database and its teams' eCoPs. *InspireNet*'s teams can be open, meaning that any *InspireNet* member may join (these are called Action Teams), or closed, meaning that only members identified by the team have access. The member database, integrated with the website, acts as a central repository for members' contact information, research interests or practice expertise, and information about members' research/quality improvement projects and grey literature. Topic-based Action Teams bring members together to develop research capacity, to foster knowledge translation and to provide a mechanism for virtual learning opportunities. Currently, Action Teams address topics of: eHealth; First Nations health; interdisciplinary public health; healthy workplace climates; new graduates' transition to professional practice; nurse educators' scholarship; palliative approach in nursing; practice-based research; students; and, a "Nursing Education and Research Rounds" team. Closed Teams use *InspireNet*'s virtual platform to bring research teams together for project collaboration using a password-protected eCoP for team members' use; currently there are 21 Closed Teams. Coordinating Teams or network-wide teams support the network itself and/or that support a function or goal common for all teams, such as a management team, advisory committee team, an evaluation team, and an annual conference planning team. Other coordinating teams serve to support members in activities across all teams, such as knowledge translation. For any of the teams, members can use their eCoP to contribute to blogs, discussion forums, an event calendar, shared document repositories, wikis and static webpages; team leaders have a link for broadcasting email to their team's members. Currently, the three types of teams, Action, Closed and Coordinating operate in a total of 58 eCoPs. The site map (Figure 2) illustrates how the website is organized; most of the site is available to the public with some areas available only to logged-in members (i.e. teams' eCoPs, database and blog comments).
